# ﻿The genus *Cacodaemon* (Coleoptera, Endomychidae) of Vietnam

**DOI:** 10.3897/zookeys.1081.75927

**Published:** 2022-01-14

**Authors:** Hiroyuki Yoshitomi, Thai Hong Pham

**Affiliations:** 1 Entomological Laboratory, Faculty of Agriculture, Ehime University, Tarumi 3-5-7, Matsuyama, 790-8566 Japan Ehime University Matsuyama Japan; 2 Mientrung Institute for Scientific Research, Vietnam National Museum of Nature, VAST, 321 Huynh Thuc Khang, Hue, Vietnam Vietnam National Museum of Nature Huynh Thuc Khang Vietnam; 3 Graduate School of Science and Technology, Vietnam Academy of Science and Technology, 18 Hoang Quoc Viet, Hanoi, Vietnam Vietnam Academy of Science and Technology Hanoi Vietnam

**Keywords:** Coccinelloidea, handsome fungus beetles, Lycoperdininae, new species, Oriental Region, taxonomy

## Abstract

The species of the genus *Cacodaemon* of Vietnam are revised. A new species, *Cacodaemonvietnamensis***sp. nov.**, is described and *C.laotinuslaotinus* (Arrow, 1920) is newly recorded from Vietnam. A previously known species, *C.proavus* Strohecker, 1964 is redescribed based on an additional female specimen and a key to species of the genus *Cacodaemon* in Vietnam is provided.

## ﻿Introduction

Lycoperdininae is the largest group of the family Endomychidae and is divided into five generic groups (Tomaszewska 2005). The *Amphisternus* group (sensu Tomaszewska 2005, 2006; = “Amphisternini” of Strohecker 1964) contains ten Oriental genera (Tomaszewska 2005, 2006; Chang and Ren 2013): *Amphisternus*, *Amphistethus*, *Blachytrycherus*, *Cacodaemon*, *Gerstaeckerus*, *Humerus*, *Ohtaius*, *Spathomeles*, *Stictomele* and *Stroheckeria*. Some of these genera have a unique characteristic of the elytra, the possession of high tubercles and/or spines (Tomaszewska 2005).

*Cacodaemon* Thomson, 1857 is an endomychid genus most famous for its unusual spiky appearance, with 26 known species/subspecies from Southeast Asia (Shockley et al. 2009). From the Indochina Subregion, only two species are recorded: *C.proavus* Strohecker, 1964 from Vietnam and *C.laotinuslaotinus* (Arrow, 1920) from Laos and China. The Sundaic species, *Cacodaemonbellicosus* (Gerstaecker, 1857), was recorded from China (Shockley et al. 2009), but this record was a misidentification of *C.laotinus* (as Amphisternusbellicosusvar.laotinus Arrow, 1920). This species should be omitted from the Chinese fauna.

In the present paper, we review the species of *Cacodaemon* known from Vietnam and describe a new species.

## ﻿Material and methods

The materials examined in this paper are preserved in the Ehime University Museum, Matsuyama, Japan (**EUMJ**), National Institute of Agrobiological Sciences, Tsukuba, Japan (**NIAS**), National Museum of Nature and Science, Tsukuba, Japan (**NSMT**), Hokkaido University Museum, Sapporo, Japan (**SEHU**), and Vietnam National Museum of Nature (**VNMN**). General observations, dissections and microstructures of dissected parts were made and photographs were taken under a Leica MZ95 stereo microscope. After observation, the dissected parts were mounted on the same card with the specimen.

Morphological abbreviations used in this study are as follows:

**EL** elytral length from anterior margin to elytral apex;

**EWH** maximum elytral width across humeral appendages;

**EWM** maximum elytral width in base of humeral appendages;

**PLM** pronotal length in median line;

**PLS** pronotal length from anterior angle to posterior margin;

**PWA** pronotal width in anterior angles;

**PWP** pronotal width in posterior angles;

**TL** total length (PLM + EL).

The average is given in parentheses after the range.

**Figure 1.**
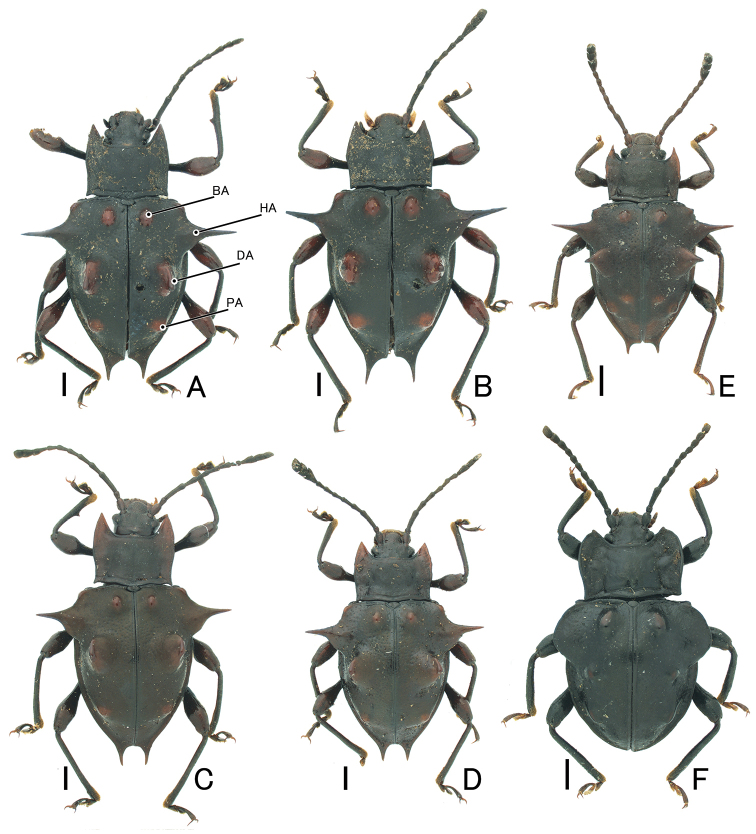
Habitus of *Cacodaemon* spp. **A–D***C.laotinuslaotinus*, Vietnamese specimens (**A, B**) and Laotian specimens (**C, D**) **E***C.vietnamensis* sp. nov., holotype **F***C.proavus***A, B** male **C, D–F** female. Scales: 1.0 mm.

Naming system and the abbreviations of elytral appendages are as follows (see also Figure 1 and Yoshitomi (2020)):

**BA** basal appendage of elytra;

**HA** humeral appendage of elytra;

**DA** discal appendage of elytra;

**PA** preapical appendage of elytra.

Morphological terminology follows Tomaszewska (2005), Yoshitomi and Sogoh (2019) and Yoshitomi (2020). The label data of the specimen examined is cited verbatim in the original spelling and given inside quotation marks (“…”).

## ﻿Taxonomy

### ﻿ Cacodaemon

Taxon classificationAnimaliaColeopteraEndomychidae

Thomson, 1857

529255C5-F8AD-52CA-8F2D-834C4E491AD4

#### Type species.

*Eumorphussatanus* Thomson, 1856 (designated by Strohecker 1964).

#### Diagnosis.

The genus *Cacodaemon* is closely related to the genus *Amphisternus* Germar, 1843, but differs from it by the following characteristics: elytral appendages spinous in most species (with tubercles or carinae in *Amphisternus*); maxillary lacinia without tuft of S-like setae at apex (present in *Amphisternus*); intercoxal process of metaventrite subparallel-sided (widening in *Amphisternus*) (after Tomaszewska 2005).

#### Biological notes.

Little is known about the ecology of *Cacodaemon* species. Adults can be collected from fungi growing on the underside of wood (Endo, personal communication).

### ﻿Key to the species of the genus *Cacodaemon* in Vietnam

**Table:** 

1	HA forming long spines, projecting laterally; DA in form of short spines	**2**
–	HA forming semicircular flat projections, projecting laterally; DA rounded	***C.proavus***
2	Smaller species, TL 7.1 mm; PA consisting of two pairs of tubercles	***C.vietnamensis* sp. nov.**
–	Larger species, TL 9.0–10.9 mm; PA consisting of one pair of tubercles	***C.laotinuslaotinus***

### ﻿ Cacodaemon laotinus laotinus

Taxon classificationAnimaliaColeopteraEndomychidae

(Arrow, 1920)

A2375B97-C35B-5682-8B59-63F249F49EB7

**Figure 2.**
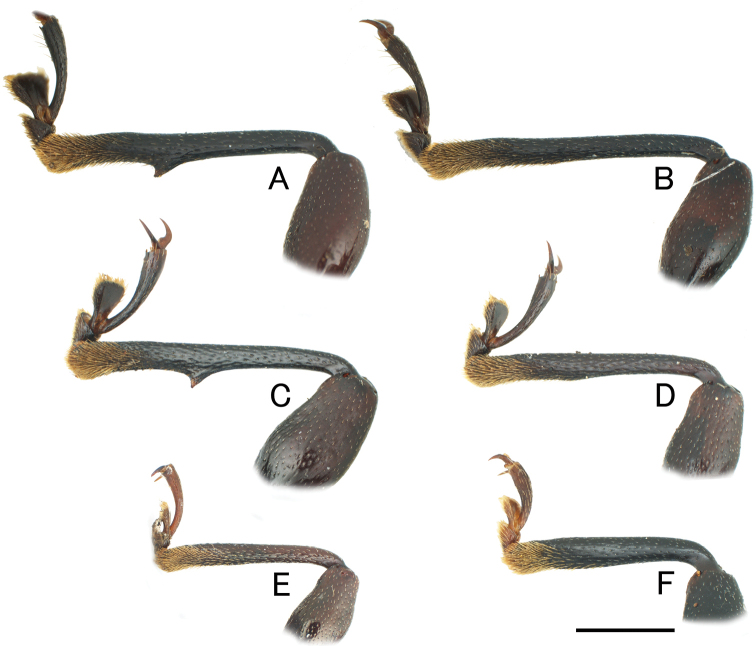
Fore tibiae of *Cacodaemon* spp. **A–D***C.laotinuslaotinus*, Vietnamese specimens (**A, B**) and Laotian specimens (**C, D**) **E***C.vietnamensis* sp. nov., holotype **F***C.proavus***A, C** male **B, D–F** female. Scale: 1.0 mm.

**Figure 3.**
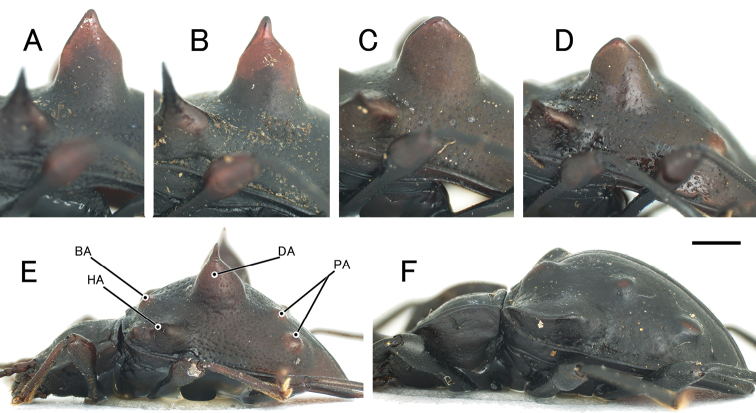
Elytral appendages of *Cacodaemon* spp. in lateral view **A–D***C.laotinuslaotinus*, Vietnamese specimens (**A, B**) and Laotian specimens (**C, D**) **E***C.vietnamensis* sp. nov., holotype **F***C.proavus***A, C** male **B, D–F** female. Scale: 1.0 mm.

**Figure 4.**
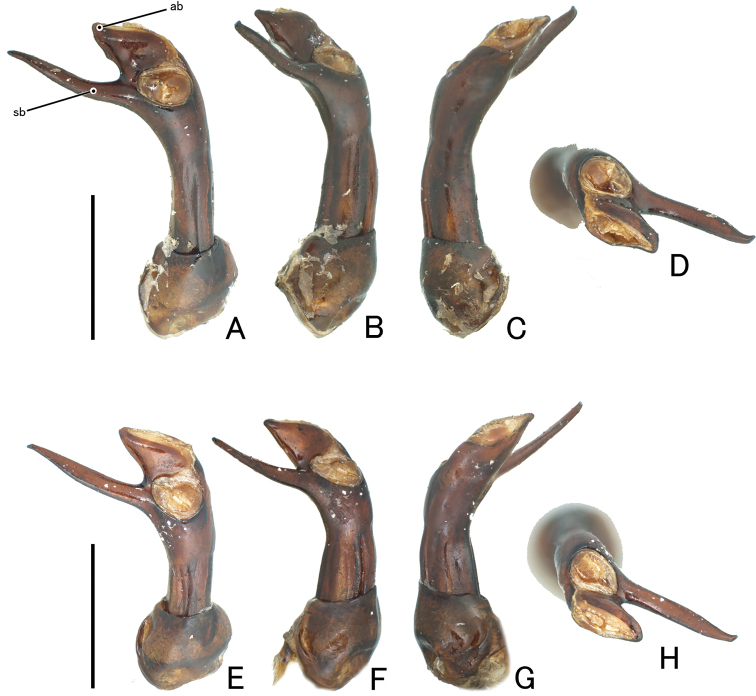
Aedeagus of *Cacodaemonlaotinuslaotinus***A–D** vietnamese specimen **E–H** laotian specimen. **A, E** dorsal **B, F** lateral **C, G** ventral **D, H** apical. Abbreviations ab: apical branch; sb: subapical branch. Scales: 1.0 mm.

**Figure 5.**
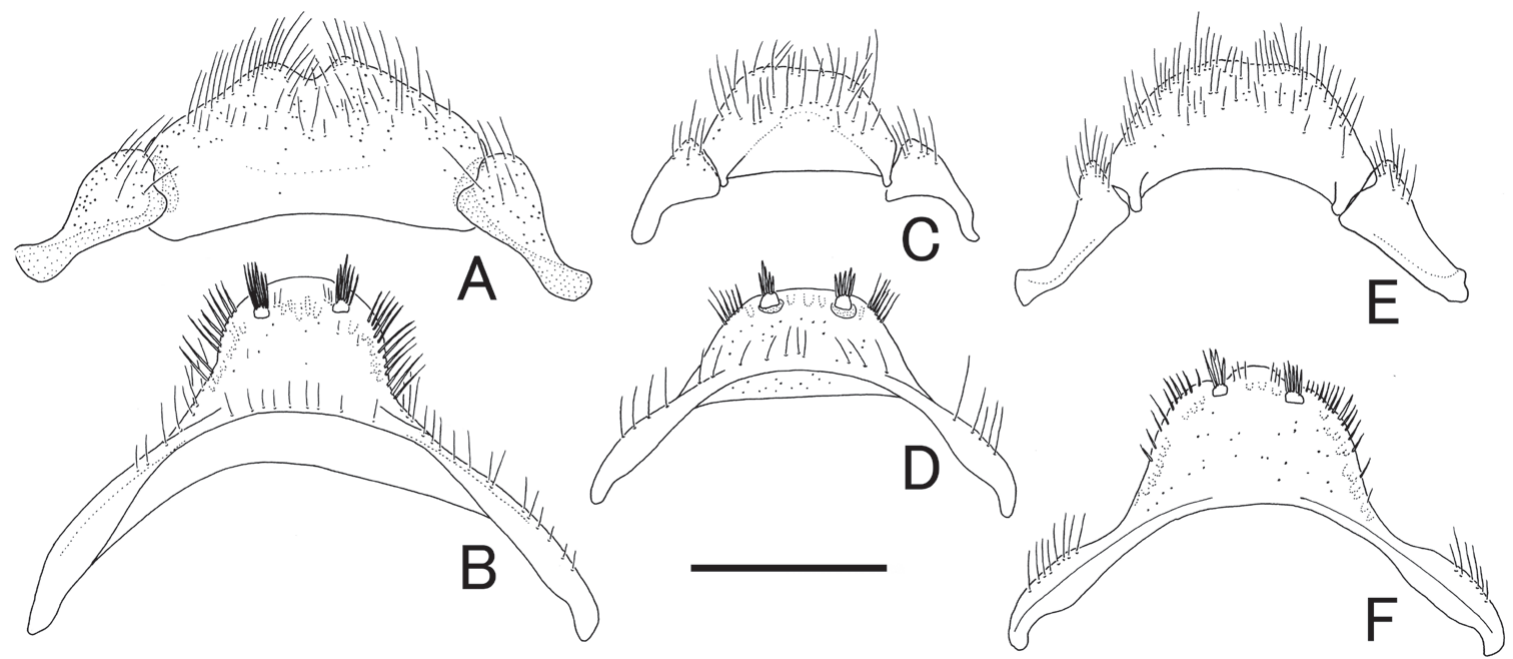
Female genitalia of *Cacodaemon* spp. **A, B***C.laotinuslaotinus***C, D***C.vietnamensis* sp. nov. **E, F***C.proavus***A, C, E** sternite VIII **B, D, F** ovipositor. Scale: 0.5 mm.

Figs 1A–1D , 2A–2D , 3A–3D , 4A–4H , 5A , 5B

Amphisternus bellicosus var. laotinus Arrow, 1920: 322.
Amphisternus laotinus : Arrow 1928: 343; Strohecker 1953: 110.
Cacodaemon laotinus : Strohecker 1964: 350; Shockley et al. 2009: 36.

#### Material examined.

1 male & 1 female (EUMJ), “[Laos] East Nong Het Xieng Khouang Prov. 25. VI. 2006 J. Yamasako leg.”; 3 males & 1 female (NIAS, VNMN), “Tam Dao N. Vietnam June 1996”, “T. Kumasawa Collection”; 1 female (NIAS), “Tam Dao N. Vietnam 1 May 1997”, “T. Kumasawa Collection”; 4 males & 1 female (SEHU), “Tam Dao VI 1994”, “A. TANAKA Coll. (田中 明) Sehu Japan 2005”.

#### Diagnosis.

This is a distinct species in the genus by having the following characteristics: elytral appendages, apical parts of femur, and antennomere 1 tinged with orange/dull orange; DA conoidal, not simply pointed.

#### Redescription.

**Male.** Body (Fig. 1A, 1C) oval, convex dorsally, weakly shiny. Coloration of body black; BA, DA, PA, humeral parts, and apical parts of femur orange; antennomere 1 I and anterolateral corners of pronotum dull orange.

Head finely punctate. Antennae long and slender; antennomere 3 longest, a little shorter than 4 and 5 combined; club (antennomeres 9–11) distinctly wide. Pronotum impunctate, microreticulate, widest at middle; basal and lateral grooves shallow; front corners projecting and pointed; lateral margins slightly tapered anteriorly and posteriorly; posterior corners slightly projecting posterolaterally; PLM/PLS 0.74–0.77 (0.75); PWM/PWA 1.14–1.23 (1.20); PWM/PLM 1.43–1.52 (1.49); PWM/PLS 1.06–1.17 (1.12). Fore tibia (Fig. 2A, 2C) straight and long, slender, with a sharp denticle at middle of inner margin. Elytra (Fig. 3A, 3C) finely and sparsely punctate, microreticulate; BA large tubercle; HA long spine, projecting laterally, stout in basal part; DA projecting dorsally, conoidal, with small spine or dull apex, stout in basal part; PA a pair of tubercles; apex of elytra with long spines; EL/EWH 0.90–0.92 (0.91); EL/PLM 3.43–3.51 (3.46); EWH/PWM 2.46–2.67 (2.58); EWH/PWM 1.73–1.67 (1.71); TL/EWH 1.15–1.19 (1.17). Aedeagus (Fig. 4) stout; apical branch (ab) short, with acute apex; subapical branch (sb) long, slightly curved, with pointed apex.

**Female** (Figs 1B, 1I, 3B, 3D). Sexual dimorphism distinct in the following characteristics: fore tibia (Fig. 2B, 2D) straight and long, lacking denticle; posterior corners of pronotum right-angled. PLM/PLS 0.73–0.74 (0.74); PWM/PWA 1.26–1.27 (1.27); PWM/PLM 1.52–1.57 (1.54); PWM/PLS 1.13–1.14 (1.14); EL/EWH 0.92; EL/PLM 3.43–3.74 (3.59); EWH/PWM 2.46–2.58 (2.52); EWH/EWM 1.76–1.72 (1.74); TL/EWH 1.17–1.19 (1.18). Sternite VIII (Fig. 5A) deeply emarginate at posterior margin. Ovipositor (Fig. 5B) bearing long setae along lateral margins of fused coxites; posterior margin of fused coxites arcuate; styli bearing long setae.

**Measurement.** Male from Vietnam (*N* = 3). TL 10.20–10.45 (10.34) mm; PWM 3.30–3.58 (3.44) mm; PWA 2.80–2.90 (2.87) mm; PLM 2.30–2.35 (2.32) mm; PLS 3.05–3.10 (3.08) mm; EL 7.90–8.10 (8.03) mm; EWH 8.80–9.00 (8.87) mm; EWM 5.10–5.40 (5.20) mm. Male from Laos (*N* = 1). TL 10.10 mm; PWM 3.45 mm; PWA 2.80 mm; PLM 2.30 mm; PLS 3.10 mm; EL 7.80 mm; EWH 8.28 mm; EWM 5.00 mm. Female from Vietnam (*N* = 2). TL 10.20–10.90 (10.55) mm; PWM 3.50–3.60 (3.55) mm; PWA 2.75–2.85 (2.80) mm; PLM 2.30 mm; PLS 3.10–3.15 (3.13) mm; EL 7.90–8.60 (8.25) mm; EWH 8.60–9.30 (8.95) mm; EWM 4.90–5.40 (5.15) mm. Female from Laos (*N* = 1). TL 9.00 mm; PWM 3.25 mm; PWA 2.70 mm; PLM 2.00 mm; PLS 3.00 mm; EL 7.00 mm; EWH 7.60 mm; EWM 4.70 mm.

#### Distribution.

Laos, Vietnam (new record).

#### Remarks.

As already mentioned by Strohecker (1964), *Cacodaemonlaotinusyunnanensis* (Kryzhanovskij, 1960) described from Yunnan (China), is thought to be an infraspecific variation. We do not treat this subspecies in this paper because the holotype could not be examined.

### ﻿ Cacodaemon vietnamensis sp. nov.

Taxon classificationAnimaliaColeopteraEndomychidae

4DA507BB-25DC-586E-BECC-8F221D501CFC

http://zoobank.org/70704EC2-3AE7-4406-9359-47E2A6898E29

Figs 1E , 2E , 3E , 5C , 5D

#### Material examined.

***Holotype***, female (EUMJ), “Bao Lac 27 km S. Vietnam 29-IV-2007 Y. YOKOI leg.”, [“Bao Lac” is probably misspelling of “Bao Loc” Lam Dong Province, S. Vietnam].

#### Diagnosis.

This species is similar to *C.laotinuslaotinus* in having conoidal DA and dull orange elytral appendages but differs from it by the following characteristics: TL smaller, PA consisting of two pairs of small tubercles.

#### Description.

**Female.** Body (Fig. 1E) oval, convex dorsally, weakly shiny. Coloration of body black; BA, DA, PA, antennomere 1 and anterolateral corners of pronotum dull orange.

Head finely punctate, closely covered with short setae. Antennae long and slender; antennomere 3 longest, a little shorter than 4 and combined; club (antennomeres 9–11) distinctly wide. Pronotum closely punctate, microreticulate, widest at apical 1/3, basal and lateral grooves deep and distinct; front corners projecting and pointed; lateral margins straight, slightly tapered posteriorly from apical 1/3; posterior corners right-angled; PLM/PLS 0.79; PWM/PWA 1.24; PWM/PLM 1.53; PWM/PLS 1.21. Fore tibia (Fig. 2E) straight and long, relatively slender. Elytra (Fig. 3E) coarsely and closely covered with shallow punctures, microreticulate; BA tubercle; HA long spine, stout in basal parts, projecting laterally; DA projecting dorsally, conoidal, with a small spine, stout in basal part; PA consisting of two pairs of small tubercles; apex of elytra with short spines; EL/EWH 0.85; EL/PLM 3.18; EWH/PWM 2.43; EWH/EWM 1.62; TL/EWH 1.12.

Sternite VIII (Fig. 5C) with tuft of short setae in postero-lateral margins of coxites; posterior margin of fused coxites gently arcuate; styli bearing short setae.

**Measurement.** Female (*N* = 1). TL 7.10 mm; PWM 2.60 mm; PWA 2.10 mm; PLM 1.70 mm; PLS 2.15 mm; EL 5.40 mm; EWH 6.33 mm; EWM 3.90 mm.

#### Distribution.

Vietnam.

#### Etymology.

Named after the type locality.

### ﻿ Cacodaemon proavus

Taxon classificationAnimaliaColeopteraEndomychidae

Strohecker, 1964

079B2E1C-8B25-532C-8E86-54CF99A0156F

Figs 1F , 2F , 3F , 5E , 5F

Cacodaemon proavus Strohecker, 1964: 346; Shockley et al. 2009: 36.

#### Material examined.

1 female (NSMT), “Mt. Pia Oac (LT: 1,200m) Cao Bang Prov. [N-Vietnam] 22. V. 1999, S. Nomura leg.”.

#### Diagnosis.

This is a distinct species in the genus by having the following characteristics: BA, DA, PA in the form of tubercles; HA carinate; apex of elytra rounded. In general appearance, this species is similar to *Amphisternussordidus* Arrow, 1928 known from Vietnam and Laos, but differs from it by the mat and smooth dorsal surface (shiny and rugose in *A.sordidus*), widely carinate HA (narrowly carinate in *A.sordidus*), and BA in the form of a tubercle (carinate in *A.sordidus*).

#### Redescription.

**Female.** Body (Fig. 1F) oval, slightly convex dorsally, weakly shiny. Coloration of body black, but DA and PA faintly dull orange.

Head moderate in size, finely punctate. Antennae long, relatively stout; antennomere 3 longest, shorter than antennomeres 4 and 5 combined; club (antennomeres 9–11) weakly widened. Pronotum indistinctly and finely punctate, microreticulate, widest at middle, widely upturned in lateral parts; front corners triangular, minutely pointed at apices; lateral margins arcuate; posterior corners right-angled; PLM/PLS 0.75; PWM/PWA 1.52; PWM/PLM 1.80; PWM/PLS 1.35. Fore tibia (Fig. 2F) straight, relatively stout. Elytra (Fig. 3F) minutely and sparsely punctate, microreticulate; basal and lateral grooves shallow; BA in the form of a tubercle; HA carinate; DA tubercle, small and low; PA tubercle, small and low; apex of elytra rounded; EL/EWH 0.99; EL/PLM 2.66; EWH/PWM 1.49; EWH/EWM 1.21; TL/EWH 1.37.

Sternite VIII (Fig. 5E) shallowly concave at posterior margin. Ovipositor (Fig. 5F) bearing short setae along lateral margins of fused coxites; posterior margin of fused coxites weakly protruding; styli bearing short setae.

**Male.** Not examined. Male genitalia figured by Strohecker (1964).

**Measurement.** Female (*N* = 1). TL 7.43 mm; PWM 3.65 mm; PWA 2.40 mm; PLM 2.03 mm; PLS 2.70 mm; EL 5.40 mm; EWH 5.43 mm; EWM 4.50 mm.

#### Distribution.

Vietnam.

#### Remarks.

Strohecker (1964) described this species based on three specimens collected from “Mauson, Tonkin” (= Mt. Mauson, Loc Binh District, Lang Son Province, Northeastern Vietnam).

## Supplementary Material

XML Treatment for Cacodaemon

XML Treatment for Cacodaemon laotinus laotinus

XML Treatment for Cacodaemon vietnamensis

XML Treatment for Cacodaemon proavus
